# A Novel Smartphone-Based Color Test for Detection of Color Vision Defects in Age Related Macular Degeneration

**DOI:** 10.1155/2022/9744065

**Published:** 2022-03-31

**Authors:** Vassilios Karampatakis, Diamantis Almaliotis, Leonidas Karamitopoulos, George Kalliris, Stavroula Almpanidou

**Affiliations:** ^1^Laboratory of Experimental Ophthalmology, School of Medicine, Aristotle University of Thessaloniki, Thessaloniki, Greece; ^2^Laboratory of Electronic Media, Aristotle University of Thessaloniki, Thessaloniki, Greece

## Abstract

**Purpose:**

To evaluate the efficacy of the smartphone-based *K*-color test to detect color defects in patients with Age-related Macular Degeneration (AMD).

**Methods:**

88 patients (*n* = 135 eyes) with AMD and 28 controls (*n* = 53 eyes) underwent color testing with the Hardy–Rand–Rittler (H-R-R), the *K*-color test, and the Ishihara test. The *K*-color test presents randomized colored shapes in decreasing steps of intensity, providing also a record system for result tele-transmission. Sensitivity, specificity, and reliability were examined to investigate the validity of the novel test. 26 participants with AMD also completed a questionnaire regarding the feasibility of the test.

**Results:**

Linear mixed-effects models indicated a significant difference (*p* < 0.001) between AMD and normal eyes. The areas under the curve (AUC) were estimated to be 0.897 [95% CI: 0.841–0.952], 0.943 [95% CI: 0.901–0.984], and 0.931 [95% CI: 0.886–0.977] for the red, green, and blue color, respectively. Based on the H-R-R, the sensitivity of the test was 0.79, 0.90, and 0.95 for the red, green, and blue colors, respectively, and specificity was 0.88 for all colors. The new test recognized more abnormal cases than the Ishihara (sensitivity of 0.98 and 1.0 and specificity of 0.48 and 0.38 for red and green colors, respectively). Test-retest reliability was found to be high for the red [ICC = 0.996 (0.990–0.999)], green [ICC = 0.974 (0.929–0.990)], and blue [ICC = 0.992 (0.981–0.997)] colors. The majority of the asked participants stated that they could easily perform the test.

**Conclusion:**

The *K*-color test was found to be sensitive and specific in detecting color defects in AMD patients. The *K*-color test may serve as a useful tool both for patients and their physicians.

## 1. Introduction

Age-related macular degeneration (AMD) is the leading cause of visual impairment in developed countries [1, 2]. Patients with AMD may lose chromatic and luminance sensitivity both in the dry and in the wet form of the disease [3–6]. Even, early changes of the anatomical integrity of the retina alter the total functionality of the cones and impair the sensitivity for processing different stimulus attributes [6]. Several aspects of cone-related visual function have been investigated in AMD patients [4, 7, 8], and the high sensitivity of the retina for detection of color signals along with the complexity of stages involved in chromatic processing make color vision testing suitable for detecting changes caused by the disease [3, 6].

The human visual system exploits the perceptual color qualities, hue, saturation, and brightness, for the identification of the environment in everyday viewing [9, 10]. The International Commission on Illumination (CIE) defines brightness (intensity) as the attribute of color sensation according to which each given visual stimulus appears more or less intense. In other words, brightness is the meter of light emittance [11–13]. There is increasing scientific interest regarding the relationship between the color-opponent mechanisms and the achromatic mechanism [11, 14–16]. There is also growing evidence that the L-M color-opponent cells in the peripheral visual pathways code achromatic intensity (brightness) as well as chromaticity [15, 17]. Color opponent neurons in the lateral geniculate nucleus (LGN) seem to respond to both changes in the chromaticity and luminance of a stimulus [18, 19]; thus, it is believed that chromatic and luminance signals are multiplexed within these cells [17]. Recent studies have shown that intermediate AMD is associated with reduced sensitivity in both color and luminance channels [3].

Readily accessible and thus commonly used color vision tests include the pseudoisochromatic plates, especially the Hardy–Rand–Rittler (H-R-R) and the Ishihara color test [20, 21]. In these tests, the chromatic contrast is embedded in luminance and spatial noise, and the subject performs a colour discrimination task [14, 22]. Though pseudoisochromatic plates have been reported to have luminous reflectance differences between the figure and background [23] and such differences alter color perception [21] and can reduce color figure detection against a gray background as luminance is changed [24, 25], in patients with cone disorders it was found that the Ishihara and H-R-R color tests were the most discriminative. Importantly, the H-R-R seemed to be the most appropriate since it allows the evaluation of all three color axes and remains accurate in participants with poor visual acuity [23]. Pseudoisochromatic plate testing is low-cost, quickly administered and therefore remains the test of choice by clinicians [25, 26].

Zarazaga et al. recommend the use of more than one color test for detecting color deficiencies; thus, there is always a need for an easy-to-perform and readily accessible color test [27]. The primary purpose of this study was to evaluate the efficacy of the smartphone-based *K*-color test, which examines intensity thresholds for colored target recognition so as to reveal subtle disturbances of color perception in AMD patients. The clinical performance and validity of the new test was also assessed in comparison with the H-R-R and the standard Ishihara color vision tests.

## 2. Methods

### 2.1. Participants

All participants were recruited from our outpatient unit at Aristotle University of Thessaloniki, School of Medicine, running the LIFE4LV program for patients with moderate and severe visual impairment and their familiars registered in the ClinicalTrial.gov (NCT05184036). We included 88 patients (*n* = 135 eyes) with AMD and 28 (*n* = 53 eyes) normal, age-matched subjects with no ocular pathology other than corrected refractive error and no known neurological disease. Informed written consent was obtained from all subjects after an explanation of the nature and possible consequences of the study. All study procedures were approved by the Committee for Bioethics and Ethics, Medical Department, Aristotle University of Thessaloniki (code#1.60/21.11.2018) and adhered to the principles embodied in the Declaration of Helsinki Code of Ethics of the World Medical Association.

### 2.2. Clinical Examination

**Figure 1:**
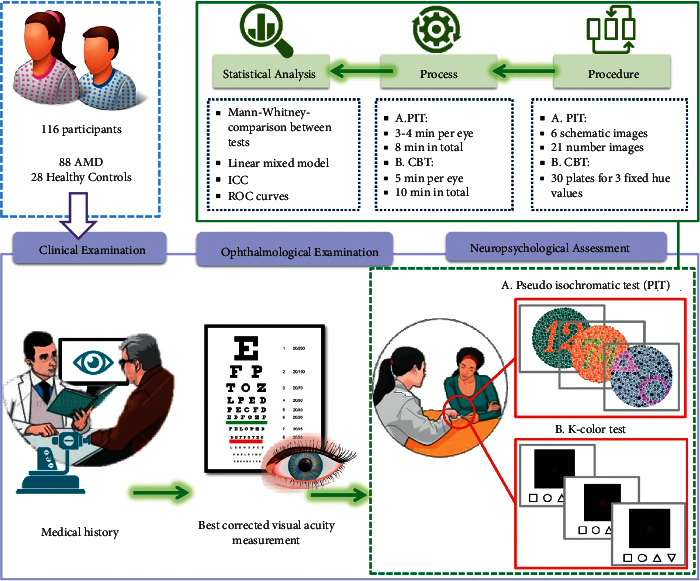
Testing procedure including a comprehensive medical history, best corrected visual acuity, and color testing. (A) Pseudoisochromatic tests, Ishihara, and H-R-R. (B) The *K*-color test with plates composed of a black square with white boundaries and a colored target in the center. Identification of the colored targets was made by selecting one shape from a choice of four that corresponds to the target in the examined plate. Statistical analysis was performed using IBM SPSS Statistics for Windows, version 26.0 (Armonk, NY: IBM Corp). ICC: intraclass correlation coefficient (95% confidence intervals); ROC curves: receiver-operating characteristic curves; CBT: color brightness test (*K*-color test).

All subjects with an established diagnosis of AMD underwent measurement of best-corrected visual acuity (BCVA) with the Early Treatment Diabetic Retinopathy Study (ETDRS) chart (Precision Vision, La Salle, Illinois, USA) under standard clinical conditions. BCVA was converted to the logarithm of the minimal angle of resolution (logMAR) visual acuity [28]. A detailed medical history was also recorded. The exclusion criteria were as follows: history of ocular pathology other than AMD, BCVA worse than 1.0 logMAR, and history of congenital color vision defects. All subjects were also free of significant lenticular changes [29, 30] and systemic conditions or medications that might have influenced the results. Color vision was evaluated monocularly by a single specialized researcher in all subjects using the H-R-R (edition 2002; Richmond Products Inc., Albuquerque, New Mexico, USA), the Ishihara Test for Color Blindness, 38 plates (edition 2018; Kanehara & Co., Ltd., Tokyo, Japan) and the new smartphone-based color test (Figure 1). Each eye of a subject was recruited separately, as earlier studies have revealed differences in color thresholds between the two eyes depending on the severity of AMD [6, 29]. However, the correlation between the left and right eyes of the same participants was taken into account in the statistical analysis.

### 2.3. Apparatus

The smartphone-based color vision test is available for devices running Android OS 5.0 or later. The application has been created with Android Studio V4.1 and the software provides various colors on the smartphone screen. The application has been demonstrated with a Samsung A30S smartphone (display: Super AMOLED, size 6.4 inches, resolution 720 × 1560 pixels, ratio 19.5 : 9, density∼268 ppi; GPU: Mali-G71 MP2) running Android OS 9.0 with maximal brightness of 489 cd/m^2^ (personal communication with Samsung Technical Support) to conduct the *K*-color vision test using full brightness screen illumination. The device was switched on at least 5 minutes before each experimental session to allow its output to stabilize.

### 2.4. Testing Procedure

The test was designed using the principles described by the hue, saturation, and value (HSV) color space model [31]. The HSV color space separates out the intensity (luminance) from the color information (chromaticity). The HSV-based approximation can determine the intensity and shade variations near the edges of a target, thereby sharpening the boundaries and retaining the color information of each pixel [31].

The selected hues for red and blue colors, which have lower inherent luminosity, corresponded to the specific wavelengths at which an individual curve can be well estimated from the luminous-efficiency [32–35].

The *K*-color test examines the thresholds of light intensity required for hue recognition which may be disrupted in patients with macular diseases [3, 4]. Both AMD participants and age-matched controls made judgements regarding the subtle changes of intensity between targets. According to Weber's law, which states that the size of the just noticeable difference (i.e., delta I) is a constant proportion of the original stimulus value, the intensity ranged from 1% to 35% for each hue value. The precision of human vision requires displays to be accurate to about 0.2% of the luminance range [36].

Each plate had a saturation of 100%, a relatively fixed hue as aforementioned, and a gradually decreasing intensity. The plates are composed of a black square with white boundaries and a colored target in the center. Hence, all stimuli were viewed against the same black surround with white boundaries to strongly suppress rod function [37], making chromaticity the only contextual parameter to intensity [38, 39]. Plates were held at approximately 40 cm away from the participants at a perpendicular angle, at the line of sight so as to avoid reflection of light. It takes approximately 5 minutes to perform the test for each eye. The total score was calculated in terms of brightness threshold (%) for target recognition.

Targets are of a size corresponding to logMAR visual acuity of 1.0, take the form of four different shapes among square, circle, triangle, and reversed triangle, and were turned at 3 seconds intervals, with a simultaneous feeling of vibration when the target changed. Each plate corresponded to a specific intensity value and was displayed four times with shapes changing randomly. On screen, instructions to the participants regarding the correct performance of the test were given. Patients used their fingers to identify the colored targets by selecting one shape from a choice of four that corresponded to the target in the examined plate (Figure 1). The answer was considered correct if the subject recognized the shape correctly three times using a forced-choice technique. The results of the test are recorded and saved in accordance with the General Data Protection Regulation (GDPR), and the physician can have access in order to evaluate the results. The test also provides users with a slider to adjust the brightness of the power display.

At the conclusion of testing, 26 participants with AMD were asked to answer a short questionnaire (Supplementary File S1) regarding the feasibility of the test and the likelihood to perform the test by themselves. The relationship of participants with new technologies was also recorded.

### 2.5. Ishihara and H-R-R Pseudoisochromatic Tests

The tests were performed monocularly and the plates were held at a perpendicular angle to the line of sight under the appropriate lighting conditions. The first screening plate of the Ishihara test, depicting the number “12,” had to be identified by all subjects. The analysis of the results was carried out according to the instructions of the test: if 4 or more errors in the first 21 plates were made, the test was classified as abnormal [40]. The H-R-R test was evaluated with the six screening plates; in case no errors were made, the test was scored as normal. The score was set as the number of plates identified out of the six screening plates (plates 5–10). Each missing number or symbol was counted as an error with an interval time of 3 seconds between plates, in both tests [41].

### 2.6. Statistical Analysis

Linear mixed-effects models were employed to determine the differences between normal and AMD eyes. Models included group of eyes (normal and AMD) and age as fixed effects. A random intercept for each participant was used to account for the correlation between the left and right eyes of the same participant. The diagnostic performance of the *K*-color test was assessed using Receiver-Operating Characteristic curves (ROCs) to estimate discriminative ability, sensitivity, and specificity for the test for varying cut-off values, and one eye from each participant was randomly selected. Reliability was evaluated by estimating the intraclass correlation coefficient (ICC) for agreement between test and retest scores and the corresponding 95% confidence intervals.

The subsequent analysis was conducted on the sample of eyes with AMD. Comparisons among the *K*-color test, the H-R-R and the Ishihara test were conducted by applying the Mann–Whitney test. The sensitivity and specificity of the *K*-color test based on H-R-R and Ishihara test results were also computed.

Statistical analysis was performed using IBM SPSS Statistics for Windows, version 26.0 (Armonk, NY: IBM Corp.). A *p* value of <0.05 was considered to indicate statistical significance.

## 3. Results

### 3.1. The Sample

The normal group consisted of 14 (50.0%) females, with an average age of 75.6 ± 11.6 years. The AMD group included eighty-eight patients; 45 (51.1%) females, with an average age of 75.8 ± 7.7 years. Demographic data for patients and controls are shown in (Table 1).

### 3.2. Comparison between AMD and Normal Eyes

Controls did not make any mistakes on the *K*-color test. Linear mixed-effects models indicated that the measured intensity thresholds in AMD eyes were significantly higher for red (*b* = −7.759, *p* < 0.001), green (*b* = −10.292, *p* < 0.001), and blue (*b* = −11.474, *p* < 0.001) targets than for age-matched normal data (*p* < 0.001) (Table 2).

### 3.3. Diagnostic Performance of the *K*-Color Test

According to ROCs analysis, the areas under the curve (AUC) were estimated to be 0.897 [95% CI: 0.841–0.952] for the red, 0.943 [95% CI: 0.901–0.984] for the green, and 0.931 [95% CI: 0.886–0.977] for the blue color (Table 3), suggesting excellent discriminative ability of the test between controls with normal color vision and AMD participants. The cut-off point (passing score) was set at 12% intensity for all colors, implying that an eye with an intensity threshold for target recognition higher than 12% was identified as an AMD eye. The sensitivity of the novel color test was 0.782 for red, 0.839 for green, and 0.839 for blue color, while specificity was 1.0 for all colors.

### 3.4. Test-Retest Reliability

Test-retest reliability was evaluated on 17 patients (*n* = 25 eyes), 9 (52.9%) of which were females, with an average age of 77.3 ± 7.6 years. The average BCVA was 0.54 ± 0.42 logMAR. Patients were examined twice within a 1-week period and their scores in the colored targets were recorded. The ICCs were high for red [ICC = 0.996 (0.990–0.999)], green [ICC = 0.974 (0.929–0.990)], and blue [ICC = 0.992 (0.981–0.997)] colors, suggesting the high repeatability and consistency of the *K*-color vision test (Table 4).

### 3.5. Comparison of the *K*-Color Vision Test Results with the Pseudoisochromatic Tests

Mann-Whitney tests showed that AMD eyes positive to the Ishihara test exhibited significantly higher intensity thresholds (%) for target recognition (*p* < 0.001) in red and green color than eyes negative to the Ishihara test, indicating that eyes positive to Ishihara needed more intensity for target recognition. Accordingly, eyes with normal H-R-R had a significantly (*p* < 0.05) lower intensity threshold for target recognition than eyes with abnormal H-R-R (Table 5).

The H-R-R test was performed in 65 eyes with AMD, and the test identified 8 (12.3%) eyes as normal and 57 (87.7%) eyes as abnormal. The sensitivity and specificity of the *K*-color test based on the H-R-R are shown on Table 6.

In the sample of AMD eyes, the Ishihara test identified 69 (51.1%) eyes as normal and 63 (46.7%) eyes as abnormal. Based on Ishihara results, the sensitivity of the novel color test in red and green colors was 0.98 and 1.0, respectively, and specificity was 0.48 and 0.38, respectively (Table 6).

### 3.6. Feedback of Feasibility

The sample consisted of 26 patients with AMD; 13 females (50, 0%) with an average age of 76.3 ± 7.9 years. Twenty patients (76, 9%) stated that the test was enough to very feasible, and sixteen patients (61.5%) stated that they used the test easily to extremely easily. Technological self-efficacy was significantly associated (rs = −0.479, *p* = 0.013) with the self-reported feasibility of the test but not with the difficulty of performing the test (rs = −0.335, *p* = 0.094). There were no differences regarding sex (feasibility: *Z* = −1,504, *p* = 0.133 and difficulty to perform: *Z* = −1,908, *p* = 0.056) and age (feasibility: rs = 0,344, *p* = 0.085 and difficulty to perform: *r*_*s*_ = 0,330, *p* = 0.099) in the examined parameters.

## 4. Discussion

In this study, we used the smartphone-based *K*-color test to evaluate color vision defects in patients with AMD. The *K*-color test was found to be reliable and it is not affected by illumination like a traditional pseudoisochromatic plate within a range of lighting conditions (avoiding ambient sunlight) since the colors are viewed on a smartphone screen [42]. This test can be used for the evaluation of blue-yellow (B-Y) vision abnormalities because it includes blue colored targets, and thus for the examination of acquired color vision deficiencies.

In the present study, we examined the intensity thresholds for colored target recognition in a sample of AMD patients and age-matched controls. The findings demonstrated that patients with AMD exhibited increased needs of intensity for red, green, and blue target recognition when compared to age-matched controls. In a recent study by Downie and colleagues, intermediate AMD was associated with reduced sensitivity in both color and luminance channels, suggesting that eyes with the same phenotype of intermediate AMD can have varying degrees of color threshold loss [3]. In the same study, relatively equal losses in each of the L-M, M-L, and S-cone mechanisms were detected [3]. Each color within the visible spectrum possesses an inherent intensity which evokes simultaneous excitation of the chromatic and the luminance channels, and this concurrent function was found to be disrupted in patients with AMD in the present study. The data from the study by Phipps et al. also supports the impact of AMD in both chromatic and luminance channels [7].

The findings from this study are indicative of disrupted function in both the B-Y and R-G axis since increased intensity thresholds were recorded for red, green, and blue recognition. This differs from some previous reports, suggesting a specific disruption in B-Y discrimination in AMD, whereas the R-G axis remains relatively intact [3, 43–45]. Other studies have found that either B-Y or R-G thresholds, or both, were abnormal in eyes with AMD, and the B-Y loss seems to be on average greater than R-G loss [7]. It is interesting that in such cases many aspects of functional vision, even visual acuity, could remain relatively normal [23, 46]. Another study supports that in most cases of acquired color vision loss, both R-G and B-Y mechanisms are disrupted with the initial loss affecting mostly B-Y discrimination and both R-G and B-Y losses are involved in the later stages of AMD [4, 29]. At the early stages of the disease, *S* cones seem to be more susceptible to changes in the metabolic environment of the retinal pigment epithelium (RPE)—photoreceptor complex [47]. One possible hypothesis to account for the specific loss of color vision is that choroidal hypoxia affects significantly the function of the highly metabolic active photoreceptors, and *S*-cones appear to be more affected [48]. These functional deficits that occur in eyes with AMD could also be attributed to the morphological alterations in Bruch's membrane and RPE, which would slow the recycling of visual photopigments [4], misalignment of the cone's outer segments causing a decrease in the optical density of the photopigment and, thus, reduced quantal catch by the photopigment [8]. In our study, both axes were found to be impaired in the vast majority of patients with AMD as assessed with all color tests, which is in line with these observations.

Several patterns of how luminance and color channels interact have been previously proposed. It is well established that *P*, *M*, and *K* ganglion cells have responses for color and luminance, but with different gains [49, 50], supporting the existence of multiple channels of luminance and color from the retina to the primary visual cortex (V1). After V1, the combination of the *M*, *P* and *K* inputs is conducted in higher order neurons with different weights [51]. Furthermore, the existence of one pathway that serves the transduction of color and luminance stimuli has not been supported by previously published data [52–54].

The most widely used global index of diagnostic accuracy is the AUC. This study included 135 eyes with AMD (positive cases) and 53 eyes of normal controls (negative cases), which is a sufficient number for ROC analysis (MedCalc, version 12.7.4; MedCalc Software, Ostend, Belgium). In general, an AUC of 0.8 to 0.9 is considered excellent, and more than 0.9 is considered outstanding [55]. In our study, ROC analysis showed that the AUC of red, green, and blue colored targets was high, indicating the high sensitivity and specificity of the *K*-color test to discriminate patients with AMD from normal controls.

We chose to detect color vision defects with the widely used in clinical practice, H-R-R and Ishihara test. In our study, participants with AMD made considerably more mistakes, predominantly on the H-R-R and on the *K*-color test. Pseudoisochromatic stimuli are composed of a combination of luminance and color contrast [56]. Most of the psychophysical studies that have combined color and luminance in the stimulus have shown that color influenced the luminance perception and vice-versa [14]. Patients with AMD were found to have abnormal testing with the H-R-R test in the majority of the affected eyes, which confirms the existence of color vision defects in these patients. Based on the Ishihara results, the *K*-color test was found to have lower specificity and equal sensitivity both in red and green colors. The low specificity is related to previous studies reported that the diagnostic performance of the Ishihara is compromised in patients with acquired color vision defects [40]. The H-R-R allows the detection of defects in the tritan axis [23, 24] and has been proved to be superior to the Ishihara test in detecting acquired color deficiencies in patients with optic neuropathy and other acquired diseases [40, 57]. We also found that increased intensity needs in patients with AMD for red, green, and blue targets are significantly associated with abnormal H-R-R and Ishihara testing.

The majority of the AMD sample experienced moderate visual impairment. In the study by Thiadens et al., visual acuity and visual field defects were not significantly associated with the severity of color vision defects [23]. In patients with cone disorders, it was found that the Ishihara and H-R-R color tests were the most discriminative, and the H-R-R seems to be the most appropriate for the evaluation of acquired deficiencies [23]. Color vision testing has been found to be accurate with the Ishihara up to 20/200 near visual acuity [58] and with the H-R-R up to 20/252 [59]. In a recent study by Almog and colleagues, the correlation between visual acuity and color vision as assessed with the Ishihara was found to be moderate in patients with macular diseases [26]. Patients with advanced age-related macular degeneration and a central scotoma may fail the Ishihara plates because of loss of central cones and yet perform correctly other color tests [26]. However, the Ishihara color vision test is widely used over other, possibly more scientific and verifiable color vision assessment tests, since it is widely available in clinical practice, it can be performed rapidly; it is very easily quantified and easy to perform for the patient [26].

With the advent of new technologies, iPhone and Android-based smartphones provide applications that may help with the management of patients with chronic diseases such as those causing low vision and are also applicable to regions deprived of easy access to medical services [60, 61]. The *K*-color test is a smartphone-based application that was able to detect defects of color vision in patients with AMD. The results can also be saved in order to be evaluated by the patient's physician. Other advantages of our test are the easy performance, as reported by some of the participants, and that the test is applicable to illiterate patients since it includes only shape recognition. Test-retest reliability was also found to be high, suggesting the high repeatability and consistency of the test. The test includes blue-colored targets which is fundamental for patients with acquired color defects. The total test time for both eyes is about 10 minutes. Moreover, as aforementioned, the colors are viewed on a smartphone screen, and therefore the *K*-color vision test is not affected by external illumination, avoiding excessive ambient lighting (sunlight) [42]. Furthermore, the patients' feedback confirmed that the *K*-color test is easily performed, even by individuals without special technological familiarity and could be suitable for self-performance, which is of high significance especially for individual with difficulties in access to healthcare facilities. Further studies are needed in order to investigate the use of the test for this purpose. Furthermore, to our knowledge, there is no sufficient evidence indicating the diagnostic performance of the pseudoisochromatic test, the Ishihara test, and the H-R-R in patients with AMD as provided by this study.

### 4.1. Limitations

Limitations of our study include the absence of comparison with the anomaloscope, which is the gold standard examination for color vision defects and with Farnsworth–Munsell 100 which is considered more sensitive in the detection of acquired color vision deficiency [42]. These tests are not easily accessible and require prolonged concentration of the patients and time of examination [62, 63].

Furthermore, we examined patients with AMD and cannot extend our findings to patients with other causes of ocular pathology. Further investigation of the diagnostic accuracy of this test in other ocular diseases is needed in order to establish its effectiveness and to evaluate its clinical usefulness. Therefore, we are currently studying a larger cohort of patients with various ocular diseases in order to expand upon our findings, and our preliminary results indicate the same efficacy. This study is a part of a wider research regarding several aspects of visual function in patients with visual impairment within the program LIFE4LV registered at ClinicalTrials.gov (NCT05184036).

The *K*-color test may serve as a complementary tool of the impact of AMD on color perception, and the ability of the test to categorize the severity of the acquired color vision deficiencies is under further investigation.

## 5. Conclusions

In conclusion, this study investigates the diagnostic performance of the smartphone based *K*-color test. The *K*-color vision test was shown to be useful for the detection of the chromatic sensitivity loss in patients with AMD in terms of intensity thresholds. The *K*-color test could serve as an adjunctive, clinical tool aiming to improve eye care accessibility, especially in underdeveloped areas which are deprived of eye care services.

## Figures and Tables

**Table 1:** Demographic data of participants.

Characteristic	AMD	Control
Number of participants	88	28
Number of eyes	135	53
Female/male	45 (51.1%)/43 (48.9%)	14 (50.0%)/14 (50.0%)
Age (years, M, SD)	75.8 ± 7.7	75.6 ± 11.6
BCVA (logMAR)	0.50 ± (0.38)	0.0 ± 0.0
Dry/wet form	93 (68, 9%)/42 (31, 1%)	—

Distribution of the clinical/demographic characteristics of the study population (age, sex, and visual acuity). BCVA = best-corrected visual acuity and logMAR = logarithm of the minimum angle of resolution.

**Table 2:** Linear mixed-effects models of the *K*-color test intensity thresholds between AMD and normal eyes.

Color	Parameter	Estimate (b)^*∗*^	95% confidence interval for b		*p* ^†^
Red	Intercept	12.775	1.914	23.636	0.022
	Group	−7.759	−11.708	−3.809	<0.001
	Age	0.108	−0.035	0.250	0.137

Green	Intercept	12.401	4.272	20.530	0.003
	Group	−10.292	−13.299	−7.284	<0.001
	Age	0.052	−0.054	0.158	0.333

Blue	Intercept	15.251	4.884	25.618	0.004
	Group	−11.474	−15.313	−7.635	<0.001
	Age	0.076	−0.060	0.212	0.268

^*∗*^ *b*: mean differences in intensity thresholds (%) for target recognition between patients with AMD and controls. ^†^*p* values <0.05 indicate statistically significant differences. Linear mixed-effects model included group of eyes and age as fixed effects, and a random intercept for participant^*∗*^.

**Table 3:** Sensitivity and specificity of the *K*-color test.

Color	AUC^*∗*^	Standard error	*p* ^†^	95% confidence interval for AUC				
				Lower	Upper	Cut-off brightness (%)	Sensitivity	Specificity
Red	0.897	0.028	<0.001	0.841	0.952	>12	0.782	1.000
Green	0.943	0.021	<0.001	0.901	0.984	>12	0.839	1.000
Blue	0.931	0.023	<0.001	0.886	0.977	>12	0.839	1.000

^*∗*^AUC: area under the receiver operating characteristic curve (ROC) for the *K*-color test scores. ^†^*p* values <0.05 indicate statistically significant discriminative ability of the test between AMD and normal eyes.

**Table 4:** Medians and quartiles of the test-retest reliability analysis.

Color	*n* (eyes)	First examination		Second examination	
		Median^*∗*^	IRQ^†^	Median^*∗*^	IRQ^†^
Red	25	27.00	18.00–31.00	27.00	17.00–31.00
Green	25	18.00	15.00–22.00	17.00	14.00–20.00
Blue	25	22.00	19.00–26.00	23.00	19.00–26.00

^*∗*^Median values indicate the lower brightness (%) needed by the 50% of the eyes. ^†^IRQ: the middle 50% of the eyes regarding the needed brightness (%) fall within the interquartile range (IRQ).

**Table 5:** Analysis of the *K*-color test results in correlation with the Ishihara and the H-R-R.

	Ishihara: normal eyes		Ishihara: abnormal eyes				
	Median^*∗*^	IRQ^†^	Median	IRQ^†^	*Z* ^‡^	*p*	Effect size

Red	14.0	11.0–23.0	29.0	24.0–31.0	−6.052	<0.001	−0.61
Green	13.0	5.0–18.0	20.0	17.3–24.0	−6.650	<0.001	−0.63

	H-R-R: normal eyes		H-R-R: abnormal eyes				

	Median^*∗*^	IRQ^†^	Median	IRQ^†^	*Z* ^‡^	*p*	Effect size

Red	11.0	11.0-11.0	24.0	11.0–29.0	−2.763	0.006	−0.38
Green	5.0	5.0-5.0	18.0	13.0–21.0	−3.809	<0.001	−0.50
Blue	8.0	8.0-8.0	21.0	19.0–26.0	−3.792	<0.001	−0.49

^*∗*^Median values indicate the lower intensity (%) needed by the 50% of the eyes. ^†^IRQ: the middle 50% of the eyes regarding the needed intensity (%) fall within the interquartile range (IRQ). ^‡^*Z*: Mann-Whitney test statistic, *p* values < 0.05 indicate statistically significant difference in brightness (%) needed between normal and abnormal eyes, effect size: shows the magnitude of the difference in brightness (%) between normal and abnormal eyes: 0.2-small, 0.5-medium, and 0.8-large.

**Table 6:** Sensitivity and specificity of the *K*-color test based on Ishihara and H-R-R results.

Novel color test	Cut-off brightness (%)	Ishihara test		H-R-R test	
		Sensitivity^*∗*^	Specificity^*∗∗*^	Sensitivity^*∗*^	Specificity^*∗∗*^
Red	>12	0.98	0.48	0.79	0.88
Green	>12	1.00	0.38	0.90	0.88
Blue	>12	—	—	0.95	0.88

^*∗*^Sensitivity is the proportion of abnormal eyes, based on Ishihara or H-R-R tests, recognized as abnormal by the test. ^*∗∗*^Specificity is the proportion of normal eyes, based on Ishihara or H-R-R tests, recognized as normal by the test.

## Data Availability

The data are available from the corresponding author on reasonable request.

## Supplementary Materials

Supplementary Materials Supplementary S1. Questionnaire-Feedback for Participants.

## References

[1] Bourne R. R. A., Jonas J. B., Bron A. M. (2018). Prevalence and causes of vision loss in high-income countries and in Eastern and central Europe in 2015: magnitude, temporal trends and projections. *British Journal of Ophthalmology*.

[2] Bressler N. M., Bressler S. B. (1995). Preventative ophthalmology. *Ophthalmology*.

[3] Downie L. E., Cheng A. S., Vingrys A. J. (2014). Color vision deficits in intermediate age-related macular degeneration. *Optometry and Vision Science*.

[4] Hogg R. E., Chakravarthy U. (2006). Visual function and dysfunction in early and late age-related maculopathy. *Progress in Retinal and Eye Research*.

[5] Liutkevičienė R., Cebatorienė D., Zaliūnienė D., Lukauskienė R., Jašinskas V. (2014). A new maximum color contrast sensitivity test for detecting early changes of visual function in age-related macular degeneration. *Medicina (Kaunas, Lithuania)*.

[6] Roopa V., Sobha S., Barbur J. L. (2017). Detection of early loss of color vision in age-related macular degeneration—with emphasis on drusen and reticular pseudodrusen. *Investigative Ophthalmology & Visual Science*.

[7] Phipps J. A., Guymer R. H., Vingrys A. J. (2003). Loss of cone function in age-related maculopathy. *Investigative Opthalmology & Visual Science*.

[8] Elsner A. E., Burns S. A., Weiter J. J. (2002). Cone photopigment in older subjects: decreased optical density in early age-related macular degeneration. *Journal of the Optical Society of America A*.

[9] Olkkonen M., Ekroll V. (2016). Color constancy and contextual effects on color appearance. *Human Color Vision*.

[10] Long F., Yang Z., Purves D. (2006). Spectral statistics in natural scenes predict hue, saturation, and brightness. *Proceedings of the National Academy of Sciences*.

[11] Blakeslee B., Reetz D., McCourt M. E. (2008). Coming to terms with lightness and brightness: effects of stimulus configuration and instructions on brightness and lightness judgments. *Journal of Vision*.

[12] Wyszecki G., Boff K. R., Kaufman L., Thomas J. B. (1986). *Bit Stealing: How to Get 1786 or More Gray Levels from an 8-bit Color Monitor1*.

[13] Wyszecki G., Stiles W. S. (1982). *Color Science: Concepts and Methods, Quantitative Data and Formulae*.

[14] Miquilini L., Walker N. A., Odigie E. A. (2017). Influence of spatial and chromatic noise on luminance discrimination. *Scientific Reports*.

[15] Andrew S., Daniela Henning P., Bruce G. (2014). Color and brightness encoded in a common *L*- and *M*-cone pathway with expansive and compressive nonlinearities. *Journal of Vision*.

[16] Caterina R., Wen L. W., Elizabeth C., Andrew S. (2009). The *S*-cone contribution to luminance depends on the *M*- and *L*-cone adaptation levels: silent surrounds?. *Journal of Vision*.

[17] Nagy A. L. (1999). Interactions between achromatic and chromatic mechanisms in visual search. *Vision Research*.

[18] De Valois R. L., Pease P. L. (1971). Contours and contrast: responses of monkey lateral geniculate nucleus cells to luminance and color figures. *Science*.

[19] Derrington A. M., Krauskopf J., Lennie P. (1984). Chromatic mechanisms in lateral geniculate nucleus of macaque. *The Journal of Physiology*.

[20] Simunovic M. P. (2016). Acquired color vision deficiency. *Survey of Ophthalmology*.

[21] Jurasevska K., Ozolinsh M., Fomins S. (2014). Color-discrimination threshold determination using pseudoisochromatic test plates. *Frontiers in Psychology*.

[22] Cormenzana Méndez I., Martín A., Charmichael T. L. (2016). Color discrimination is affected by modulation of luminance noise in pseudoisochromatic stimuli. *Frontiers in Psychology*.

[23] Thiadens A. A. H. J., Hoyng C. B., Polling J. R., Bernaerts-Biskop R., van den Born L. I., Klaver C. C. W. (2013). Accuracy of four commonly used color vision tests in the identification of cone disorders. *Ophthalmic Epidemiology*.

[24] Huna-Baron R., Glovinsky Y., Habot-Wilner Z. (2013). Comparison between Hardy-Rand-Rittler 4th edition and Ishihara color plate tests for detection of dyschromatopsia in optic neuropathy. *Graefe’s Archive for Clinical and Experimental Ophthalmology*.

[25] Zhao J., Davé S. B., Wang J., Subramanian P. S. (2015). Clinical color vision testing and correlation with visual function. *American Journal of Ophthalmology*.

[26] Almog Y., Nemet A. (2010). The correlation between visual acuity and color vision as an indicator of the cause of visual loss. *American Journal of Ophthalmology*.

[27] Fanlo Zarazaga A., Gutiérrez Vásquez J., Pueyo Royo V. (2019). Review of the main colour vision clinical assessment tests. *Archivos de la Sociedad Espanola de Oftalmologia*.

[28] Khoshnood B., Mesbah M., Jeanbat V., Lafuma A., Berdeaux G. (2010). Transforming scales of measurement of visual acuity at the group level. *Ophthalmic and Physiological Optics*.

[29] O’Neill-Biba M., Sivaprasad S., Rodriguez-Carmona M., Wolf J. E., Barbur J. L. (2010). Loss of chromatic sensitivity in AMD and diabetes: a comparative study. *Ophthalmic and Physiological Optics*.

[30] Nagai K., Sasaki H., Jonasson F. (2009). Who cataract classification system and cataract surgery in-12 years. *Investigative Ophthalmology & Visual Science*.

[31] Sural S., Gang Qian G., Pramanik S. Segmentation and histogram generation using the HSV color space for image retrieval.

[32] Ayama M., Ikeda M. (1998). Brightness-to-luminance ratio of colored light in the entire chromaticity diagram. *Color Research & Application*.

[33] Ikeda M., Shimozono H. (1978). Luminous efficiency functions determined by successive brightness matching. *Journal of the Optical Society of America*.

[34] Yaguchi H., Kawada A., Shioiri S., Miyake Y. (1993). Individual differences of the contribution of chromatic channels to brightness. *Journal of the Optical Society of America A*.

[35] MacLeod D. I. A., Boynton R. M. (1979). Chromaticity diagram showing cone excitation by stimuli of equal luminance. *Journal of the Optical Society of America*.

[36] Tyler C. W. (1997). Colour bit-stealing to enhance the luminance resolution of digital displays on a single pixel basis. *Spatial Vision*.

[37] Barbur J. L., Stockman A. (2010). Photopic, mesopic and scotopic vision and changes in visual performance. *Encyclopedia of the Eye*.

[38] Lotto R. B., Clarke R., Corney D., Purves D. (2011). Seeing in colour. *Optics & Laser Technology*.

[39] Barbur J. L., Rodriguez-Carmona M., Harlow A. Establishing the statistical limits of “normal” chromatic sensitivity.

[40] Cole B. L., Lian K. y., Lakkis C. (2006). The new Richmond HRR pseudoisochromatic test for colour vision is better than the Ishihara test. *Clinical and Experimental Optometry*.

[41] Bailey J. E., Neitz M., Tait D. M., Neitz J. (2004). Evaluation of an updated HRR color vision test. *Visual Neuroscience*.

[42] Shin Y. J., Park K. H., Hwang J.-M. (2014). A novel color vision test for detection of diabetic macular edema. *Investigative Opthalmology & Visual Science*.

[43] Collins M. J. (1986). Pre‐age related maculopathy and the desaturated D‐15 colour vision test. *Clinical and Experimental Optometry*.

[44] Haegerstrom-Portnoy G., Brown B. (1989). Two-color increment thresholds in early age related maculopathy. *Clinical Vision Sciences*.

[45] Applegate R. A., Adams A. J., Cavender J. C., Zisman F. (1987). Early color vision changes in age-related maculopathy. *Applied Optics*.

[46] Roopa V., Sobha S., Barbur John L. (2016). A study of chromatic sensitivity loss in age related maculopathy (ARM) with emphasis on reticular drusen, soft drusen and other ARM morphologies. *Investigative Ophthalmology & Visual Science*.

[47] Spraul C. W., Lang G. E., Grossniklaus H. E., Lang G. K. (1999). Histologic and morphometric analysis of the choroid, Bruch’s membrane, and retinal pigment epithelium in postmortem eyes with age-related macular degeneration and histologic examination of surgically excised choroidal neovascular membranes. *Survey of Ophthalmology*.

[48] Barbur J. L., Connolly D. M. (2011). Effects of hypoxia on color vision with emphasis on the mesopic range. *Expert Review of Ophthalmology*.

[49] Lee B. B., Sun H., Valberg A. (2011). Segregation of chromatic and luminance signals using a novel grating stimulus. *The Journal of Physiology*.

[50] Dacey D. M., Lee B. B. (1994). The “blue-on” opponent pathway in primate retina originates from a distinct bistratified ganglion cell type. *Nature*.

[51] Conway B. R. (2014). Color signals through dorsal and ventral visual pathways. *Visual Neuroscience*.

[52] Mullen K. T., Losada M. A. (1994). Evidence for separate pathways for color and luminance detection mechanisms. *Journal of the Optical Society of America A*.

[53] Stromeyer C. F., Kronauer R. E., Ryu A., Chaparro A., Eskew R. T. (1995). Contributions of human long-wave and middle-wave cones to motion detection. *The Journal of Physiology*.

[54] Mullen K. T., Cropper S. J., Losada M. A. (1997). Absence of linear subthreshold summation between red-green and luminance mechanisms over a wide range of spatio-temporal conditions. *Vision Research*.

[55] Faraggi D., Reiser B. (2002). Estimation of the area under the ROC curve. *Statistics in Medicine*.

[56] Mollon J. D., Shevell S. K. (2003). *The Origins of Modern Color Science in the Science of Color*.

[57] Matti A. I., Chu E. R., Keane M., Pesudovs K., Celia C. S. (2011). Comparison of Ishihara and Hardy-Rand-Rittler pseudoisochromatic plates in non-arteritic anterior ischaemic optic neuropathy. *Neuro-Ophthalmology*.

[58] Chen A. A. (2011). The effect of decreased near visual acuity on ishihara’s test for color blindness. *Investigative Ophthalmology & Visual Science*.

[59] McCulley T. J., Golnik K. C., Lam B. L., Feuer W. J. (2006). The effect of decreased visual acuity on clinical color vision testing. *American Journal of Ophthalmology*.

[60] Ozgur O. K., Emborgo T. S., Vieyra M. B., Huselid R. F., Banik R. (2018). Validity and acceptance of color vision testing on smartphones. *Journal of Neuro-Ophthalmology*.

[61] Han X., Scheetz J., Keel S. (2019). Development and validation of a smartphone-based visual acuity test (vision at home). *Translational Vision Science & Technology*.

[62] Zabel J., Przekoracka-Krawczyk A., Olszewski J., Michalak K. P. (2021). Variability of Rayleigh and moreland test results using anomaloscope in young adults without color vision disorders.. *PLoS One*.

[63] Birch J. (2008). Failure of concordance of the Farnsworth D15 test and the Nagel anomaloscope matching range in anomalous trichromatism. *Visual Neuroscience*.

