# Supplementary material for: A Novel Smartphone-Based Color Test for Detection of Color Vision Defects in Age Related Macular Degeneration
Source: J Ophthalmol. 2022 Mar 31;2022:9744065. doi: 10.1155/2022/9744065 (PMC8991385; doi:10.1155/2022/9744065)
Supplement: Supplementary Materials — Supplementary S1. Questionnaire-Feedback for Participants. [file 9744065.f1.docx]

**Questionnaire-Feedback for Participants**

*Script:*

Thank you for agreeing to take part in this study. This survey will ask you about your views on the K-color test.

All responses that you provide will remain confidential and no identifying information will be published. This survey is expected to take approximately 3 minutes.

**Participant ID: ____________________**

**Date: ____________________**

1. How feasible do you find the K-color test?
   - Very feasible
   - Feasible enough
   - Neither feasible nor inappropriate
   - Less feasible
   - Not feasible at all
2. How difficult would it be to perform the K-color test by yourself?
   - Extremely easily
   - Easily
   - Neutral
   - Difficult
   - Extremely difficult
3. What is your relationship with new technologies (smartphones etc.)?
   - Excellent
   - Good
   - Neutral
   - Bad
   - Very bad
